# Supplementary material for: Effect of a social media-based health education program on postnatal care (PNC) knowledge among pregnant women using smartphones in Dhulikhel hospital: A randomized controlled trial
Source: PLoS One. 2023 Jan 20;18(1):e0280622. doi: 10.1371/journal.pone.0280622 (PMC9858435; doi:10.1371/journal.pone.0280622)
Supplement: S1 Appendix — (DOCX) [file pone.0280622.s002.docx]

## **S2: Intervention protocol**

**Title:** **Effect of a social media-based health education program on postnatal care (PNC)knowledge among pregnant women using smartphones in Dhulikhel hospital: A Randomized Controlled Trial**

**Objectives of the research:**

General objective: To assess the effect of a social media-based health education program on PNC knowledge among pregnant women attending Dhulikhel Hospital.

Specific objective(s):

To assess the effect of a social media-based health education program on PNC knowledge score when compared to usual care among pregnant women in Dhulikhel hospital

**Participants:** Literate pregnant women owning a smartphone and visiting Dhulikhel hospital for ANC checkups will be enrolled in the study. Pregnant women must have mobile applications like Viber, What's the app, and Facebook messenger for being eligible for the study. Pregnant women having vision impairment or learning difficulties such as dementia will be excluded from the study.

**Intervention:** The intervention includes a 16-minute PNC video containing general information on postnatal care including frequency, timing, and place providing postnatal care; danger signs of mother and newborn; different services provided at each PNC visit, and the importance of PNC visit. The PNC video is based on the Health Belief Model designed to provide tailored information on postnatal care.

Age

Marital status

Ethnicity

Religion

Education

Occupation

Family size

Income

Access to media

Obstetric factors:

- Gestational week
- Parity
- Gravida
- ANC frequency
- Planned pregnancy

Change in PNC knowledge

Cues to action:

Self-motivation and self-diagnosis of danger signs through illustrated images in video

Perceived Severity

Consequences and risk of not having PNC visit

Perceived benefits of PNC visit

Information on advantages and importance of PNC visit

Perceived barriers of PNC visit

Perceived self-efficacy

Perceived threat

Fig 1.1 Conceptual framework based on Health Belief Model

**Procedure:**

Step 1: The researcher will register pregnant women meeting eligibility criteria for the study. We will gather information for the interactive messaging software such as gestational age, mobile phone number, and instant messaging software application during registration from the gynecology OPD in Dhulikhel hospital.

The inclusion criteria of the participants in the social media-based study includes currently pregnant women at third trimester, owns a smartphone with social media (What's app, Viber, Facebook), has internet connectivity at home or mobile data and can read and write Nepali.

The exclusion criteria are:

1. Women who have learning difficulties (dementia)
2. vision impairment

The principal researcher will check for the eligibility of the registered pregnant women. The investigator and trained research assistant will take verbal consent of the eligible pregnant women through telephone interview and enroll in the study.

Step 2: We will randomize pregnant women providing consent to either intervention or usual care arm in 1:1 ratio through a random sequence number generated by STATA 14 using simple randomization as per the random table number.

Step 3: The investigators will conduct telephonic interviews to collect baseline information using the Kobo toolbox. The link of the kobo toolbox questionnaire for baseline and knowledge assessment is mentioned below:

Registration: https://ee.kobotoolbox.org/x/Mfpkh0kT

Baseline: https://ee.kobotoolbox.org/x/NScq22E3

Step 4: We will send PNC educational video message to pregnant women assigned to the intervention group either through Viber, What's App, or Facebook messenger. Facebook is the most popular social media in Nepal. It is a widely used platform for chatting, sharing media, creating groups, and making new friends. So, we will use this social media to send educational PNC videos to pregnant women. We will use alternative instant messenger such as Viber and What's App if the pregnant women are not Facebook users. The principal researcher will create a new account on Facebook on the name of the study and communicate the account name and password to the Research Assistants. We will use a single Facebook account to send video messages as well as follow up the pregnant women for video views. We will also create a group of women enrolled in the intervention for interaction regarding PNC video. However, for alternatives to this social media (Viber and What's App) we will use our personal Viber and What's app number for sending video messages and monitoring the video views.

Before sending the PNC video message to pregnant women in the intervention group, we will call and inform the pregnant women about the PNC video and provide a brief message on PNC visit in Dhulikhel hospital; request pregnant women to view the educational video at least 4 times from the enrollment till 4 weeks before delivery. The script for making a call before sending the PNC video is mentioned in Annex - I.

PNC Video: https://www.youtube.com/watch?v=_cJxjcJ9eIc

The women in the control group will receive the usual care. The pregnant women assigned to usual care will not receive any active intervention.

Step 5: We will follow up with pregnant women for the frequency of PNC video views through telephone calls as well as Facebook messages, and send the reminder message to pregnant women every week to view the video. The pregnant women will view the video at least 4 times after enrolment in the intervention. We will ensure whether the pregnant women viewed the PNC educational video by sending messages on Facebook. We will also make calls to pregnant women if we do not receive a response to the messages in Facebook messenger. We will also track the frequency of PNC video views by monitoring the views of the video on YouTube.

Step 6: We will collect information on change in PNC knowledge after ensuring the recommended PNC video views. After 4 times of PNC video views in 1 month of intervention, we will make calls to the pregnant women to assess change in PNC knowledge. We will use a structured Kobo toolbox questionnaire to record women's responses electronically. The link of the kobo toolbox questionnaire is mentioned below:

https://ee.kobotoolbox.org/x/M43SET3u

We will create a Google sheet to record and monitor the calls, remainder, and video views. This Google sheet will serve as the tracking system of the respondents.

The summary of the intervention is mentioned in Fig 1.


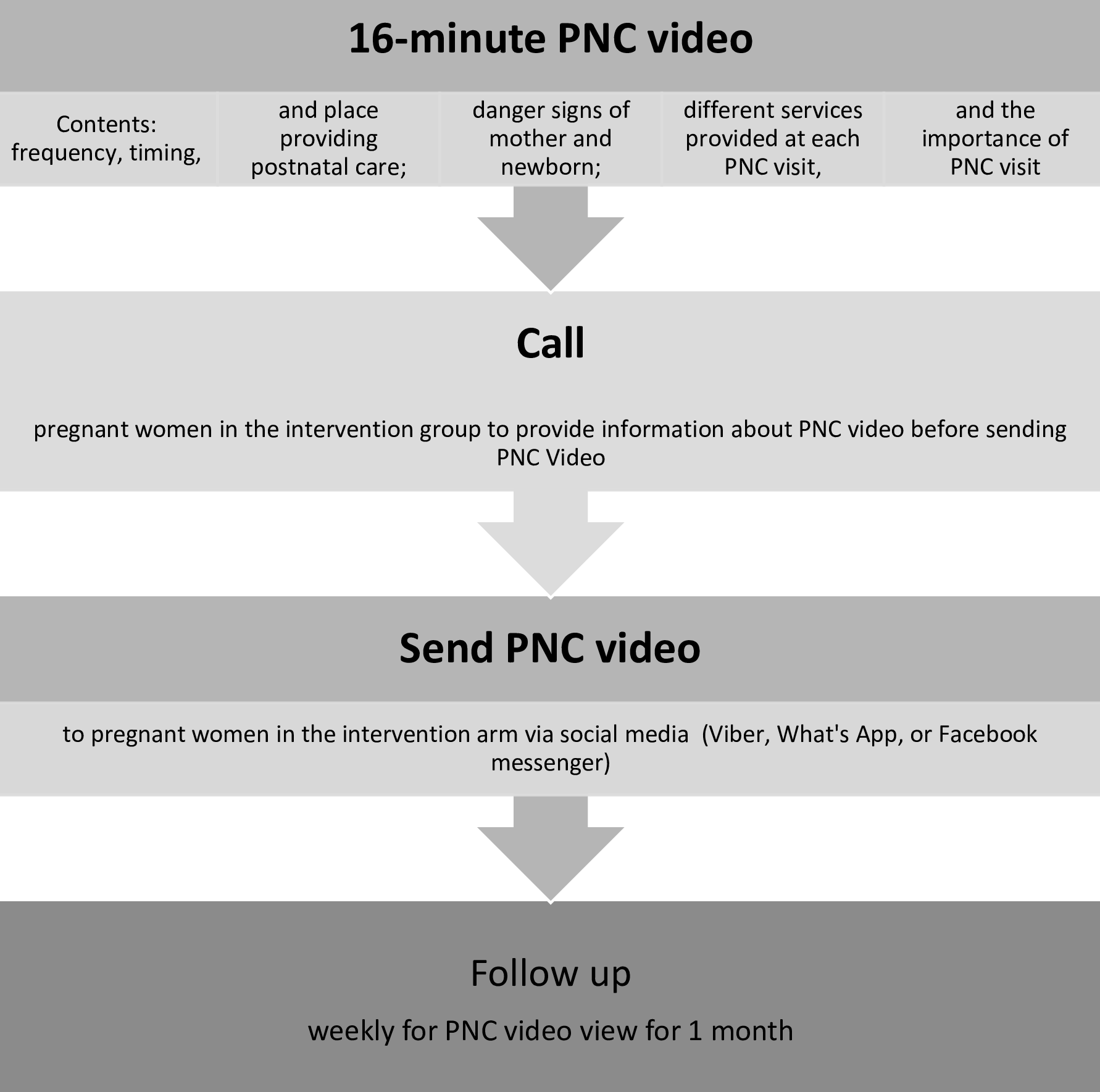


Fig 1.2: Intervention matrix

**Annex I: Script to call for PNC video message**

नमस्ते म ………………………..काठमाण्डौ विश्वबिद्यालय स्कुल अफ मेडिकल साइन्सबाट बोल्दैछु ।  आज म तपाईंलाई सुत्केरी शैक्षिक सामाग्रीको भिडियो पठाउन लागेको कुरा जानकारी गराउन फोन गरेको हो । गर्वभती जाँचका लागि धुलिखेल आउने स्मार्ट फोन प्रयोग गर्ने गर्वभती महिलामा सुत्केरी स्याहारको ज्ञान र सेवा उपायोग सम्वन्धी अध्ययनमा तपाईं आवद्द हुनुहुन्छ । सोही अध्ययनको लागि यो शैक्षिक भिडियो पठाउदैछु । यो सुत्केरी भिडियोमा सुत्केरी स्याहार भनेको के हो, किन गर्नुपर्छ, यसका के के फाईदा छन, सुत्केरी अवस्थामा देखिने खतराका लक्षण के के हुन इत्यादी बारे जानकारी दिन्छ । यो जानकारीले सुत्केरी महिलाको सुत्केरी सम्बन्धी ज्ञान बढाउन मद्दत गर्नुका साथै आफ्नो र नवजात शिशुको उचित स्याहार गर्नमा सहयोग हुन्छ ।  तपाईंले यो भिडियो अध्ययन अवधिभर कम्तिमा पनि ४ पटक अनिबार्य हेर्न अनुरोध गर्दछु। । तेसपछी सुत्केरी सम्बन्धी  ज्ञानको मुल्याङ्कन गर्न अन्तर्वाता लिनेछौ । अन्त्यमा तपाईको अमुल्य समय र सहयोगको लागि धेरै धेरै धन्यवाद ।

Translation of the script

Namaste! I am……(name)………. from Kathmandu University School of Medical Sciences. Today, I am making call to inform you that you are enrolled in a study entitled “Effect of a social media-based health education program on postnatal care (PNC)knowledge among pregnant women using smartphones in Dhulikhel hospital: A Randomized Controlled Trial”. We are sending you PNC video in your social media as a part of the study. This PNC video contains information on what is PNC, need of PNC visit, benefits of PNC visit, services provided during PNC visit, danger signs of mother and newborn during postpartum period and timing for making PNC visit. The information on PNC will generate awareness on PNC and develop confidence of mother in taking care of themselves and their babies. I request you to view this video at least 4 times. After that we will make your knowledge assessment. Lastly, thank you for your valuable time and consideration.
