# Supplementary figures and images for: Effect of a social media-based health education program on postnatal care (PNC) knowledge among pregnant women using smartphones in Dhulikhel hospital: A randomized controlled trial
Source: PLoS One. 2023 Jan 20;18(1):e0280622. doi: 10.1371/journal.pone.0280622 (PMC9858435; doi:10.1371/journal.pone.0280622)

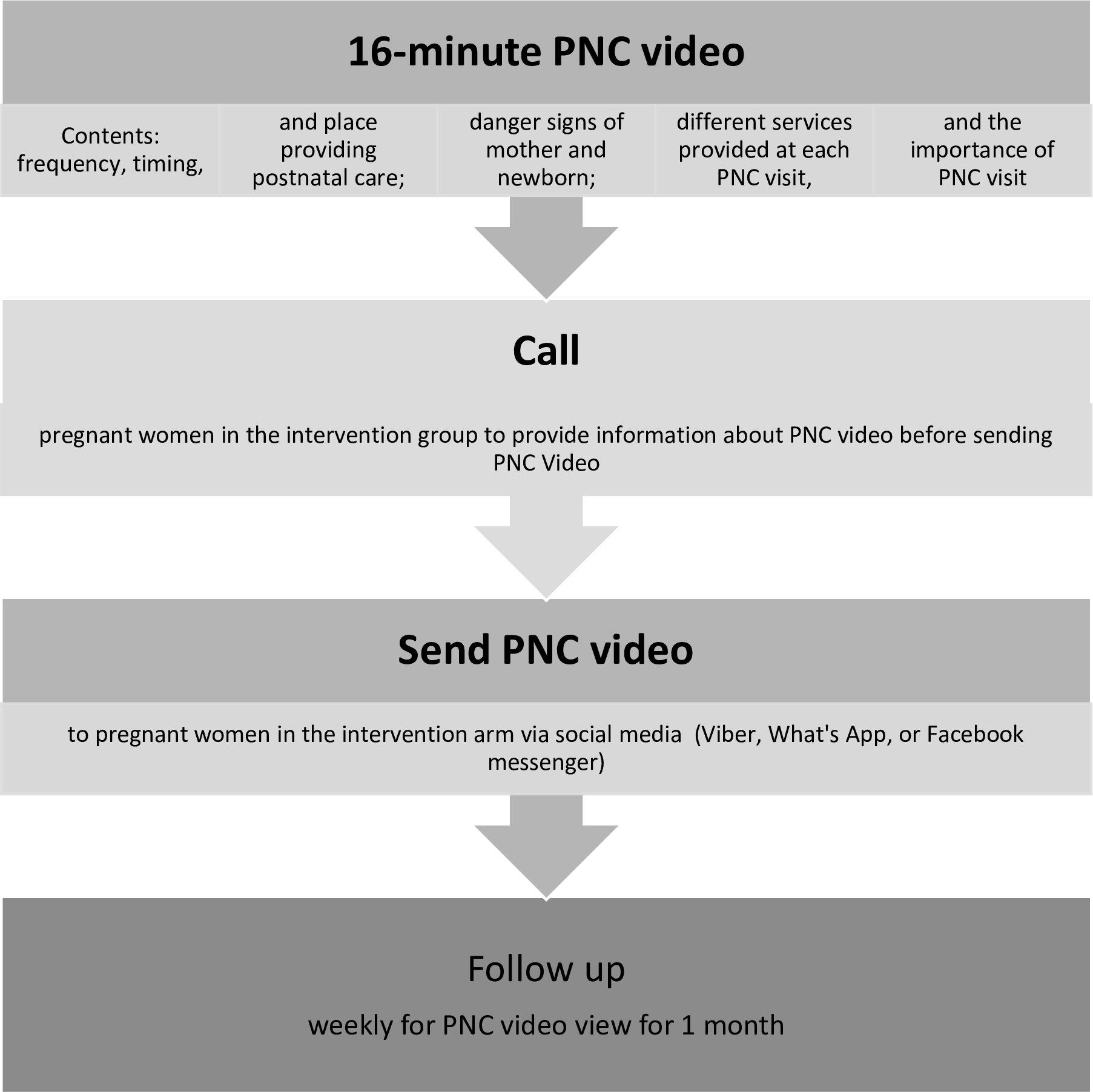

Supplement: S1 Fig — (TIF) [file pone.0280622.s003.tif]

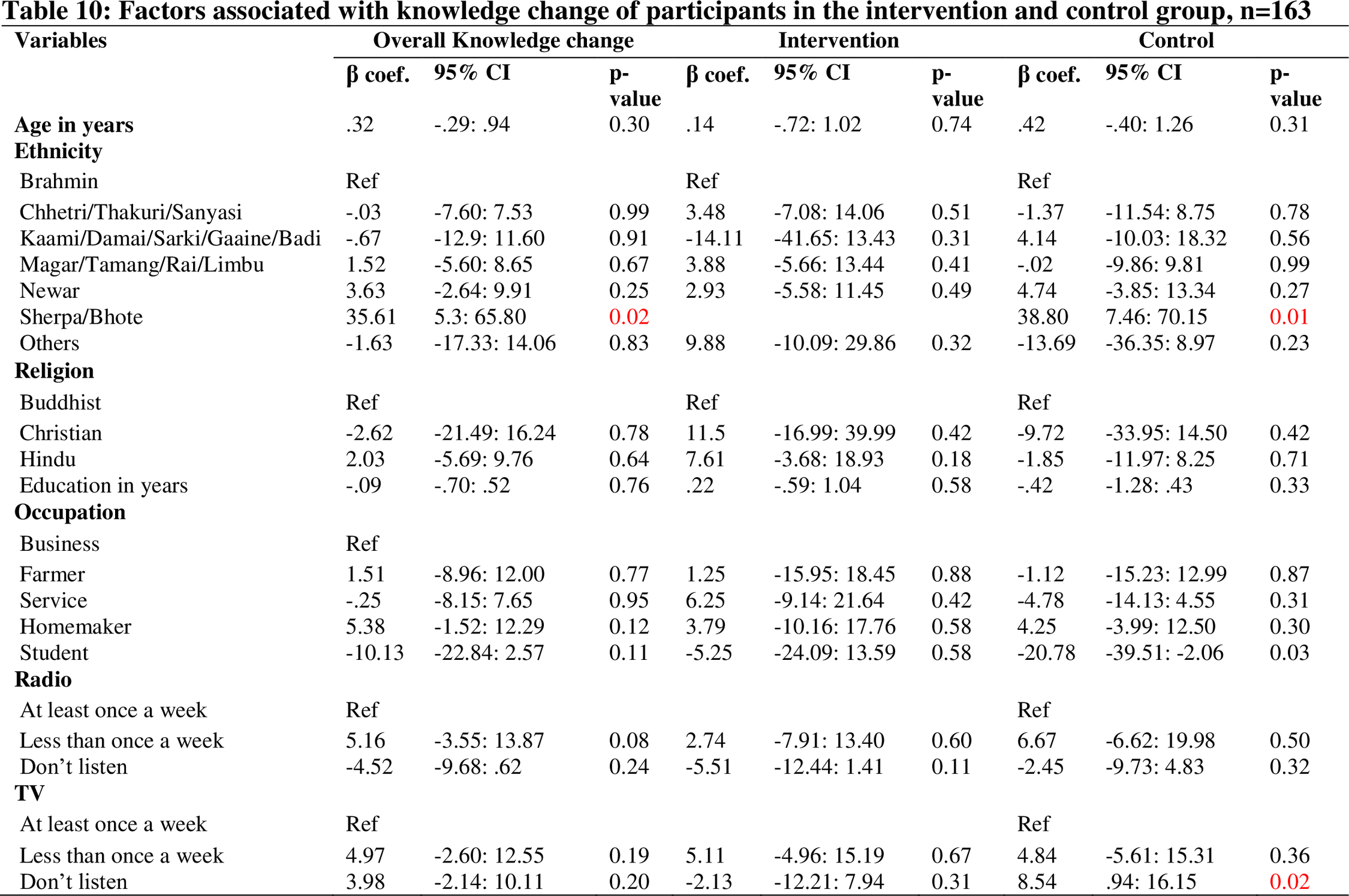

Supplement: S1 Table — (TIF) [file pone.0280622.s004.tif]
