# Supplementary material for: Effect of a social media-based health education program on postnatal care (PNC) knowledge among pregnant women using smartphones in Dhulikhel hospital: A randomized controlled trial
Source: PLoS One. 2023 Jan 20;18(1):e0280622. doi: 10.1371/journal.pone.0280622 (PMC9858435; doi:10.1371/journal.pone.0280622)
Supplement: Attachment — Submitted filename: Response to Reviewers.docx [file pone.0280622.s006.docx]

October 10, 2022

Editorial Office

PLOS ONE

Dear Editor,

It is with enthusiasm that I resubmit a revised version of the manuscript titled “Effect of a Social media-based Health Education Program on Postnatal care (PNC) Knowledge among Pregnant Women using Smartphones in Dhulikhel Hospital: A Randomized Controlled Trial”.

Thank you for giving us the opportunity to revise and resubmit this manuscript. I appreciate the time and effort provided by the reviewer. I have incorporated the suggested changes into the manuscript to the best of my ability. The manuscript has certainly benefited from these insightful suggestions. I look forward to working with you and the reviewer to move this manuscript closer to publication in the Journal of PLOS ONE.

Append to this letter is our response to the comments raised by the reviewer and the editor. As

you will notice, we agreed with all the comments raised by the reviewers. Accordingly, we have uploaded a copy of the original manuscript marked with all the changes in track change made during the revision process, as well as the revised clean version. We have also uploaded the manuscript data as a supplemental file along with this revised file.

Sincerely yours,

Kalpana Chaudhary

Kathmandu University School of Medical Sciences

Dhulikhel, Kavre, Nepal

Email: kalpana298@gmail.com

Phone no: + 977 9840983993

**RESPONSE TO REVIEWER**

**Reviewer #1: The article has clearly reported the findings that are significant in addressing maternal and child health, a major global public health concern. All the section are well articulated. Therefore the paper can be accepted after the minor corrections are done.**

**We appreciate your comment. Thank you.

Comments to the authors
Title: Clear and specific to the concept under study
Abstract: The introduction statement on background (Postnatal care (PNC)” can detect and subsequently manage life threatening complications” (need to be rephrased since PNC is a service and it can’t detect or manage rather it is through the service that this can be done.**

We rephrased the introduction statement in the abstract. The rephrased statement in the abstract in line 32 is: Postnatal care services help in detecting and subsequently managing life threatening complications.

**Methods: Looks okay, but since the ratio of allocating group was 1:1, then the numbers should not have differed much 109 vs 120. Results and conclusion are well articulated.**

Despite the allocation ratio of 1:1, the numbers in the control and intervention arm differed due to incomplete enrollment of the study participants due to COVID 19 pandemic. We described the reasons for unequal enrollment in the methods section of the manuscript as:

The 1:1 allocation was not equal. We could not enroll the original target sample size of 296 as enrollment had to be paused due to COVID-19 pandemic, leading to discrepancies in the participant's allocation to control(n=120) or intervention(n=109). However, this sample size had enough power (more than 90%) to assess the effect of social media-based education on PNC knowledge. [18,33]

References:

18.	Shiferaw S, Spigt M, Tekie M, Abdullah M, Fantahun M, Dinant G-J. The Effects of a Locally Developed mHealth Intervention on Delivery and Postnatal Care Utilization; A Prospective Controlled Evaluation among Health Centres in Ethiopia. PLoS One. 2016;11: e0158600. doi:10.1371/journal.pone.0158600

33.	Beraki GG, Tesfamariam EH, Gebremichael A, Yohannes B, Haile K, Tewelde S, et al. Knowledge on postnatal care among postpartum mothers during discharge in maternity hospitals in Asmara: A cross-sectional study. BMC Pregnancy Childbirth. 2020;20. doi:10.1186/s12884-019-2694-8

**Introduction: The section has well described background of the area under the study. Grammatical errors such as in the year 2017 instead of in 2017. Low- and middle-income countries can be abbreviated. Delete the word resulting in this sentence “Nepal resulting from pregnancy complications or childbirth in 2016”.**

In line 68, we corrected the grammatical error as in the year 2017 instead of in 2017. We abbreviated low-and middle-income countries as LMIC in line 70. In line 73, we deleted the word resulting in the sentence. Thank you for noting it.

**Line 96: The statement need revision “Several social media-based program like Facebook, Twitter embraced across the globe to strengthen knowledge and delivery of maternal, neonatal, and child health services”.**

We revised the statement as: Several social media-based program like Facebook, Twitter embraced across the globe to strengthen knowledge have shown optimistic results in the delivery of maternal, neonatal, and child health services.[17–22]

References:

17.	Feroz A, Perveen S, Aftab W. Role of mHealth applications for improving antenatal and postnatal care in low and middle income countries: a systematic review. BMC Health Serv Res. 2017;17: 704. doi:10.1186/s12913-017-2664-7

18.	Shiferaw S, Spigt M, Tekie M, Abdullah M, Fantahun M, Dinant G-J. The Effects of a Locally Developed mHealth Intervention on Delivery and Postnatal Care Utilization; A Prospective Controlled Evaluation among Health Centres in Ethiopia. PLoS One. 2016;11: e0158600. doi:10.1371/journal.pone.0158600

19.	Lund S, Nielsen BB, Hemed M, Boas IM, Said A, Said K, et al. Mobile phones improve antenatal care attendance in Zanzibar: a cluster randomized controlled trial. BMC Pregnancy Childbirth. 2014;14: 29. doi:10.1186/1471-2393-14-29

20.	Maslowsky J, Frost S, Hendrick CE, Cruz FOT, Merajver SD. Effects of postpartum mobile phone-based education on maternal and infant health in Ecuador. Int J Gynaecol Obstet. 2016;134: 93. doi:10.1016/J.IJGO.2015.12.008

21.	Shorey S, Lau Y, Dennis CL, Chan YS, Tam WWS, Chan YH. A randomized-controlled trial to examine the effectiveness of the ‘Home-but not Alone’ mobile-health application educational programme on parental outcomes. J Adv Nurs. 2017;73: 2103–2117. doi:10.1111/jan.13293

22.	Ajay, Cunningham K, Manandhar S, Shrestha N, Chen M, Weissman A. Exploring the Use of Mobile Health to Improve Community-Based Health and Nutrition Service Utilization in the Hills of Nepal: Qualitative Study. J Med Internet Res 2020;22(9)e17659 https//www.jmir.org/2020/9/e17659. 2020;22: e17659. doi:10.2196/17659

**Line 103: Correct the word improved to improve.**

We corrected the word improved to improve.

**Line 103: remove the word subsequently, since this is not in Pakistan which is the country referred to in the preceding statement.**

We deleted the word subsequently.

**Methods: The section is well elaborated but there is need to correct grammatical errors. Line 147, learning difficulties (dementia), this can be written as, such as dementia, putting it in brackets gives this a different meaning.**

We removed the bracket and revised the statement as: The women were excluded if they were identified with learning difficulties such as dementia or vision impairment.

**Line 172:correct the word reminder.**

We corrected the word reminder.

**Line 173; remove “the” in before the making.**

We removed “the” in before the making.

**Line 203, remove “a” in “relevant a guideline”.**

We removed “a” in “relevant a guideline”.

**Line 219 add the content for (1 item).**

We added “where to go if experience danger signs for (1 item)” in line 231.

**Line 222, remove 2in” from “in the”.**

We removed “in the” from line 222.

**Line 225-226, need editing for the statement “The total sum was 226 computed one during the recruitment and the other at the end of the study”. Though Health belief model is referred to in this study, it is not clear how it was used and how the its components were related or integrated into the concept of social media and PNC. Apart from ethical clearance, were there other ethical considerations. How was rigor maintained.**

We revised the statement as: The total sum was computed twice: (1) at enrollment and (2) at the end of the study.

The health belief model was used to develop the contents of the PNC video with the aim of not just increasing knowledge but also promoting health related behavior change. We have added the health belief model conceptual framework and additional clarification as follows: Health belief model hypothesizes that people will take action to prevent illness if they consider themselves susceptible to a particular health condition. People tend to change health behaviors if they believe particular health condition has grave consequences in their health. We used HBM to develop intervention with the aim of making pregnant women aware of perceived susceptibility and perceived severity of postnatal care which in turn increases perceived self-efficacy. [34] (Fig 1)

Reference:

34.	Glanz K, Rimer B k., Viswanath K. HEALTH BEHAVIOR AND HEALTH EDUCATION Theory, Research, and Practice 4TH EDITION. 4th Editio. Glanz K, Rimer B k., Viswanath K, editors. Jossey-Bass; 2002. Available: https://www.academia.edu/6487965/HEALTH_BEHAVIOR_AND_HEALTH_EDUCATION_Theory_Research_and_Practice_4TH_EDITION

Age

Marital status

Ethnicity

Religion

Education

Occupation

Family size

Income

**Access to media: PNC educational video**

**Obstetric factors:**

-Gestational -week

-Parity

-Gravida

-ANC frequency

-Planned pregnancy

Change in PNC knowledge

Individual behavior:

PNC service utilization

Cues to action:

Self-motivation and self-diagnosis of danger signs through illustrated images in video

Perceived Severity

Consequences and risk of not having PNC visit

Perceived benefits of PNC visit

Information on advantages and importance of PNC visit

Perceived barriers of PNC visit

Perceived self-efficacy

Perceived threat

Figure 1. Conceptual Framework based on the Health Belief Model

We interviewed participant at separate room to maintain privacy. The nature of the participation was voluntary. Any form of coercion to answer the question was forbidden.

**Results: The section is well done analysis and good presentation of the findings. In the consortium flow diagram, it is not clear why wrong number is tabulated twice in follow up.**

We corrected the CONSORT flow diagram.

In total, the number of participants lost to follow up were 2 and is now tabulated at one place only. Thank you for pointing that out.

**Discussion: Line 413, I suggest you change the statement hence pass the critical postpartum successfully to go through the postpartum period successfully.**

We corrected the statement as: The increased maternal and newborn care attributes knowledge develops confidence among women to take care of themselves and their babies, adapt to new conditions and to go through the postpartum period successfully and also increase PNC service utilization.

**Line 460 (The univariate analysis reported social media-based education significantly associated with their PNC knowledge score) is not clear.**

We removed the statement “The univariate analysis reported social media-based education significantly associated with their PNC knowledge score” as we have not reported univariate results in the manuscript.

**The discussion has only compared findings with similar setting highlighting Tanzania, Indonesia and India.**

In the discussion section, we only compared the findings with Tanzania, Indonesia and India because we could not find similar studies done in other countries. We added one more study conducted in Iran in the discussion section with the following text:

In a study by Parisa, the mean health literacy score after the intervention increased by 12.68 ± 6.31(mean score=58.03 ± 6.57) score in the experimental group and 0.21 ± 4.25 (mean score=42.23 ± 8.47) in the control group from 45.35 ± 6.75 and 42.02 ± 7.41 score in the experimental and control group respectively at baseline. Compared to the present study, higher change in the mean knowledge score after mobile app training was attributed by the duration of the intervention (8 weeks) and the nature of the intervention where the participants viewed the educational material in the mobile app, and also had access to researcher’s cellphone number to contact if she had any questions about the app. Also, the change in mean knowledge score in the intervention group was influenced by age of women and spouse, education level of women and spouse, occupation of women and spouse, monthly income, and place of residence. [53]

References:

53.	Karamolahi PF, Bostani Khalesi Z, Niknami M. Efficacy of mobile app-based training on health literacy among pregnant women: A randomized controlled trial study. Eur J Obstet Gynecol Reprod Biol X. 2021;12: 100133. doi:10.1016/J.EUROX.2021.100133

**The study in India does not elaborate on what type of intervention, therefore not clear if they are comparable.**

In the study done in India, the intervention included audio messages highlighting nutrition, iron/folic acid supplementation, calcium supplements, development of the fetus, ANC reminders, anemia, rest during pregnancy, blood tests, HIV testing, sonography, danger signs during pregnancy, birth preparedness, identifying labor pains, cutting the umbilical cord/dressing the stump, family planning, sanitation and hygiene, breastfeeding practices, colostrum, and sex determination. These messages were sent two times in a week during pregnancy. So, we compared our study with this study as both studies aim to see the change in knowledge. We also added the following text in the discussion to elaborate how it was comparable:

In contrast to the findings from our study, pseudo randomized controlled trial with similar intervention (audio messages included messages on maternal and new born care; and messages were sent twice a week) and conducted in India among 2016 pregnant women found no significant changes in knowledge of pregnant women in the intervention group.

**The references need editing since some are incomplete and wrongly done.**

We revised the incomplete and wrongly done references.

**Reviewer #2: This manuscript presents a randomized controlled trial to measure the effectiveness of a social media-based Health Education Program on Postnatal Care (PNC) among Nepali women. Postnatal care is often considered as a neglected reproductive health service in many countries which highly influences the morbidity and mortality rate among new mothers and newborns. Therefore, this manuscript falls into an area where more research is highly needed and appreciated.

In an attempt to promote awareness and PNC education among pregnant women, a number of pregnant women have participated in the intervention that consists of watching a 16-min video elaborated by the authors on different aspects of PNC throughout a period of one month. The authors state that the participants in the intervention group received weekly reminders to watch the video via phone calls. The control group, on the other hand, was given the usual antenatal care.

Overall, the manuscript is well-written and well-structured. However, there are multiple weaknesses on this study, particularly in terms of the intervention approach.**

We appreciate positive comments from the reviewer.  **First, I cannot discern the usefulness of having to watch the ‘same non-changing’ video on a weekly basis during that one-month of intervention.**

The main purpose of having to watch “the same non-changing” video on a weekly basis during the one-month intervention was to reinforce learning by repetition. Pregnant women may not grasp the main message from the video while watching the video in a single view. Based on the health education principle, repetitive view of the educational video would result in retention and persistence of the knowledge.

We have added the clarification in the manuscript as follows: Based on the health education principle, repetitive view of the educational video would result in retention and persistence of the knowledge.[38]

References:

38.	HHS: Principles Of Health Education. [cited 10 Oct 2022]. Available: http://ecoursesonline.iasri.res.in/mod/page/view.php?id=20743

**Also, how did you make sure that the video has been watched more than once?**

We have added this in the methods section: We assessed if the participants watched the video by asking the participants on weekly telephone calls for four weeks, relying on self-report.

We acknowledge that self-report is a limitation of the study, so added it in the limitation section with the following text: Fourth, the assessment of video views by pregnant women was self-reported. However, we tracked the number of views on the YouTube -- there were about 701 views on the Youtube videos for 167 intervention participants.

**The issue in this study is that we do not get the real objective of the study; whether is it measuring the effectiveness of promoting PNC awareness through a video or measuring the efficacy of the video to disseminate information about PNC? I believe that the authors are akin to promote PNC education but I think the approach adopted is quite weak and inadequate.**

This study reported the efficacy of the video on disseminating information about PNC resulting in the improvement in PNC service knowledge. We have added the following text in the background to provide further rationale and clarification of the study objective:

We aim to test the hypothesis that a social media-based information dissemination on postnatal service will improve postnatal care knowledge among pregnant women. Improved awareness and knowledge on postnatal care could lead to improved PNC practices at household and community level, with a potential to lower maternal deaths [26] and increase newborn survival. [19,27]

References:

19.	Lund S, Nielsen BB, Hemed M, Boas IM, Said A, Said K, et al. Mobile phones improve antenatal care attendance in Zanzibar: a cluster randomized controlled trial. BMC Pregnancy Childbirth. 2014;14: 29. doi:10.1186/1471-2393-14-29

20.	Maslowsky J, Frost S, Hendrick CE, Trujillo Cruz FO, Merajver SD. Effects of postpartum mobile phone-based education on maternal and infant health in Ecuador. Int J Gynecol Obstet. 2016;134: 93–98. doi:10.1016/j.ijgo.2015.12.008

21.	Shorey S, Lau Y, Dennis CL, Chan YS, Tam WWS, Chan YH. A randomized-controlled trial to examine the effectiveness of the ‘Home-but not Alone’ mobile-health application educational programme on parental outcomes. J Adv Nurs. 2017;73: 2103–2117. doi:10.1111/jan.13293

22.	Ajay, Cunningham K, Manandhar S, Shrestha N, Chen M, Weissman A. Exploring the Use of Mobile Health to Improve Community-Based Health and Nutrition Service Utilization in the Hills of Nepal: Qualitative Study. J Med Internet Res 2020;22(9)e17659 https//www.jmir.org/2020/9/e17659. 2020;22: e17659. doi:10.2196/17659

23.	Feroz A, Rizvi N, Sayani S, Saleem S. Feasibility of mHealth intervention to improve uptake of antenatal and postnatal care services in peri-urban areas of Karachi: a qualitative exploratory study. J Hosp Manag Heal Policy. 2017;1: 5–5. doi:10.21037/jhmhp.2017.10.02

24.	Carmichael SL, Mehta K, Srikantiah S, Mahapatra T, Chaudhuri I, Balakrishnan R, et al. Use of mobile technology by frontline health workers to promote reproductive, maternal, newborn and child health and nutrition: a cluster randomized controlled Trial in Bihar, India. J Glob Health. 2019;9. doi:10.7189/JOGH.09.020424

25.	Lavender T, Richens Y, Milan SJ, Smyth RMD, Dowswell T. Telephone support for women during pregnancy and the first six weeks postpartum. Cochrane Database Syst Rev. 2013. doi:10.1002/14651858.CD009338.pub2

26.	Oyeyemi SO, Wynn R. The use of cell phones and radio communication systems to reduce delays in getting help for pregnant women in low- and middle-income countries: a scoping review. Glob Health Action. 2015;8: 28887. doi:10.3402/gha.v8.28887

27.	Crehan C, Kesler E, Nambiar B, Dube Q, Lufesi N, Giaccone M, et al. The NeoTree application: developing an integrated mHealth solution to improve quality of newborn care and survival in a district hospital in Malawi. BMJ Glob Heal. 2019;4. doi:10.1136/BMJGH-2018-000860

**Another issue worth mentioning is the use of the Health Belief Model. This model was developed to help understand and predict health behaviors. I do not see its applicability in the present study. The authors reported that the video has been designed using the HBM in a way that the video would likely highlight the risks and benefits that new mothers will get during their postnatal period. The authors are advised to review the utilization of HBM.**

We have added further rationale and clarification of using HBM as follows: We used the Health Belief Model to design the educational video because the Health Belief Model (HBM) is a common and widely used health education approach to explain and predict preventive behaviors. HBM has been previously used to design educational intervention for improvement of oral health behavior in grade-schoolers [35] and maternal education. [36]

According to the HBM as applied to PNC education, when mothers find themselves susceptible to PNC problems and understand the severity of such problems, they will be more likely to adopt recommended PNC related health behaviors. Other elements of the model, such as the balance between perceived threat and benefits, how people react to media-perceived action cues, and how well people believe they can avoid disease, all help people change harmful behaviors and adopt healthy ones. Here, PNC service utilization is referred to healthy behaviours. We plan to make follow up postpartum women to see the effect of PNC video on service utilization in coming days.[37]

References:

35.	Sanaeinasab H, Saffari M, Taghavi H, Karimi Zarchi A, Rahmati F, Al Zaben F, et al. An educational intervention using the health belief model for improvement of oral health behavior in grade-schoolers: a randomized controlled trial. BMC Oral Health. 2022;22: 1–11. doi:10.1186/S12903-022-02132-2/TABLES/3

36.	Khani Jeihooni A, Mohammadkhah F, Razmjouie F, Harsini PA, Sedghi Jahromi F. Effect of educational intervention based on health belief model on mothers monitoring growth of 6–12 months child with growth disorders. BMC Pediatr 2022 221. 2022;22: 1–10. doi:10.1186/S12887-022-03593-8

37.	Mbuthia F, Reid M, Fichardt A. mHealth communication to strengthen postnatal care in rural areas: a systematic review. BMC Pregnancy Childbirth. 2019;19. doi:10.1186/S12884-019-2531-0

We also added conceptual model and further clarification on it, as mentioned above.

**Plus, according to the statistical results provided, there is no substantial effectiveness perceived in raising awareness about PNC among the participants through the video’s content. The analysis shows quite similar results between the control and intervention groups.**

We have added further explanation in the discussion section as follows:

The total PNC knowledge score increased by 8 points among intervention group compared to control group, which is 23% higher score among intervention group compared to control group. The maternal care attribute score was increased by 4.3 points, which is 18% higher score in intervention group compared to control group; and newborn care attribute score was increased 3.39 points, which is 32% higher in intervention group compared to control group.

**I sincerely value the time and effort deployed in this study and I do recommend the authors to exploit the results they have obtained differently to give more strength to their study. A comparative analysis between participants in terms of demographics or education or other criteria, could be valuable.**

We incorporated factors associated with knowledge of participants in the intervention and control group as a supplemental table(S8_Table 10) in this manuscript.

**Reviewer #3: A two-arm randomized controlled clinical trial was conducted which aimed to assess the effect of an intervention of a social media-based health education program on postnatal care knowledge among pregnant women at a single institution in Nepal. Postnatal care knowledge score, maternal care attribute knowledge, and newborn care significantly increased in the intervention arm compared to the control arm.

Minor revisions:
1- Carefully proofread the abstract, paying attention to the placement of commas.**

We have revised the abstract. Thank you for pointing this out.

**2- The standard statistical term for average is mean.**

We have changed the word average to mean.

**3- State and justify the study’s target sample size with a pre-study statistical power calculation. The power calculation should include: (1) the estimated outcomes in each group; (2) the α (type I) error level; (3) the statistical power (or the β (type II) error level); (4) the target sample size and (5) for continuous outcomes, the standard deviation of the measurements.**

We have added the justification in the manuscript as follows:

The sample size calculation was made as: a total of 296 women, 148 in each arm were calculated to detect an effect size of 2(OR) between the intervention and control arm with 59 % of women having their PNC visit within 7 days of delivery. The sample size calculation was based on the assumption of 80% power and 5 % significance level with an attrition rate of 10 percent. The sample size was calculated using Cliniccal Software. [31,32]

References:

31.	Sample Size Calculator. 2020. Available: https://clincalc.com/stats/samplesize.aspx

32.	Rosner B, Bernard A. Fundamentals of biostatistics. Thomson-Brooks/Cole; 2006.

**4- Line 240: Replace “in” with “as.”**

We replaced “in” with “as” in line 240. Thank you for noting it.

**5- Indicate if the continuous variables presented in the tables were checked for normal distributions prior to applying the t-tests.**

We have checked the variables for normal distribution which is already mentioned in the line 274 as follows:

The visual inspection of these graphs showed the data were linear, normally distributed and had equal variance.

**6- Lines 367-8: Provide measures of dispersion for the values 38.02 and 37.31.**

We included the measures of dispersion values as follows:

The overall PNC knowledge score in the intervention group was 38.02(**±**12.75) and, in the control, group was 37.31(**±**13.53) at baseline.

**7- Bar graph figures: Label the y-axes.**

We labelled the y-axis of the bar graph.

**8- Figure S3: Overall is one word.** **Knowledge is misspelled.**

We corrected spellings in Figure S3.
